# Microstructure and mechanical properties of hard *Acrocomia mexicana* fruit shell

**DOI:** 10.1038/s41598-018-27282-8

**Published:** 2018-06-25

**Authors:** E. A. Flores-Johnson, J. G. Carrillo, C. Zhai, R. A. Gamboa, Y. Gan, L. Shen

**Affiliations:** 1 CONACYT – Unidad de Materiales, Centro de Investigación Científica de Yucatán, Calle 43, No. 130, Chuburná de Hidalgo, Mérida, 97205 Yucatán Mexico; 2 Unidad de Materiales, Centro de Investigación Científica de Yucatán, Calle 43, No. 130, Chuburná de Hidalgo, Mérida, 97205 Yucatán Mexico; 3 School of Civil Engineering, The University of Sydney, NSW, 2006 Australia; 4 Instituto Tecnológico Superior de Motul, Carretera Mérida-Motul, Tablaje Catastral 383, Motul de Carrillo Puerto, 97430 Yucatán Mexico

## Abstract

Fruit and nut shells can exhibit high hardness and toughness. In the peninsula of Yucatan, Mexico, the fruit of the Cocoyol palm tree (*Acrocomia mexicana*) is well known to be very difficult to break. Its hardness has been documented since the 1500 s, and is even mentioned in the popular Maya legend The Dwarf of Uxmal. However, until now, no scientific studies quantifying the mechanical performance of the Cocoyol endocarp has been found in the literature to prove or disprove that this fruit shell is indeed “very hard”. Here we report the mechanical properties, microstructure and hardness of this material. The mechanical measurements showed compressive strength values of up to ~150 and ~250 MPa under quasi-static and high strain rate loading conditions, respectively, and microhardness of up to ~0.36 GPa. Our findings reveal a complex hierarchical structure showing that the Cocoyol shell is a functionally graded material with distinctive layers along the radial directions. These findings demonstrate that structure-property relationships make this material hard and tough. The mechanical results and the microstructure presented herein encourage designing new types of bioinspired superior synthetic materials.

## Introduction

Natural materials can exhibit outstanding mechanical properties that are often superior to those of their constituents^1^. These remarkable properties found in biological materials from both plants^2,3^ and animals^4^ are the result of evolutionary developments and architecture optimization leading to high-performance lightweight materials, made of relatively weak and mundane constituents^5,6^, with complex hierarchical structure and topography^5^. Classic examples of natural materials with exceptional performance of mechanical properties made of limited constituents include seashells, wood, bamboo, bone and teeth^7–10^. In recent years, the study of biological materials to understand their mechanical properties and microstructure is growing at a fast rate^11^ with the aim to develop sources of inspiration for the design of superior synthetic materials^12–15^. Bioinspired material design brings the possibility of developing new multifunctional materials with diverse technological applications^1,16,17^. However, a deep understanding towards structure-property relationships at different length scales, to unveil the underlying governing mechanisms and design principles of biological materials, is needed^16,18^.

Plants are an excellent source of inspiration for biomimetic materials^19,20^ because they can exhibit desirable mechanical properties such as high bending stiffness and toughness^21–23^, impact resistance^24^, and hierarchical multifunctional structures^25^. Recent studies have been focusing on the mechanical performance and microstructure of the hard shell of nuts^26^ and fruit seeds^27^, which are known to protect the seed against predators^27^, for the development of impact-resistant and energy-absorbing materials^28^. Some studies have been performed to measure the force required to break nut and fruit shells in order to understand fracture behaviour^29–32^; however, fracture resistance may be influenced by the size and shape of the shell^33^ and thus more studies are needed to understand the intrinsic mechanical properties of the material. More complete studies of the endocarp of Macadamia nuts to understand the high toughness and hardness observed in the shell of this biomaterial have been performed^31^. It was found that the hierarchical cellular structure is responsible for its good mechanical performance^34–36^. Nanoindentation of the seed coats of tropical plants have shown high hardness and a complex hierarchical structure in these materials^37^.

The Cocoyol or Coyol (*Acrocomia mexicana* Karw. ex Mart.^38^) is a tropical palm tree found in the Yucatan Peninsula in Mexico from the family Arecaceae. In recent years, the fruit of this palm (as *Acrocomia aculeata*) has been extensively studied because of the great potential for the production of solid biofuel^39,40^ due to its high content of cellulose and lignin^41^. The hardness of Cocoyol endocarp (as *Acrocomia aculeata*) has been mentioned only anecdotally in the scientific literature^42–44^ and in personal narrative books^45,46^. The hardness of the Cocoyol endocarp was first documented by the Spanish priest Diego de Landa in 1566^45^, who described it as a very hard kernel that the Maya people had to open with a stone to reach the edible part. There even exists a wild Maya legend called “The Dwarf of Uxmal”^46,47^, recounted by J. L. Stephens in 1882 during his visit to the Maya ruins of Uxmal^48,49^, in which the Cocoyol hardness is mentioned. In this legend, the Dwarf of Uxmal was challenged by the Governor’s city who told him to collect two bundles of “Cocoyol, a fruit of a very hard species,” with one of which he, the Governor, would beat the Dwarf over the head, and afterwards the Dwarf should beat him with the other. The Governor thought that the Dwarf would die before it was his turn in the challenge; however, the Dwarf survived the challenge and the Governor died when the Cocoyol fruits were smashed in his head. The spectators hailed the Dwarf as the new Governor of Uxmal.

While some parts of the legend are very questionable, the questions that remain out of curiosity are: Is the Cocoyol fruit very hard indeed? Are the recollections of Diego de Landa’s book about the hardness of the Cocoyol accurate? Until now, no scientific studies quantifying the hardness of the Cocoyol endocarp have been found in the literature to prove or disprove that this fruit is indeed “very hard”. In the authors’ preliminary work on the Cocoyol fruit^50^, it was found that the required force (normalized to the shell thickness) to break the whole Cocoyol fruit subjected to compression was about 1.4 kN/mm, which is around 50% higher than the force reported for similar size fruit and nut shells^29,30,36^. These preliminary findings and the references to the Cocoyol hardness found in several anecdotal works motivated our curiosity to prove (or disprove) that the Cocoyol is indeed a “very hard” fruit; this warrants a quantitative investigation to underpin the structural origins of this enhanced hardness. In this work, this is addressed with an extensive study of the microstructure and mechanical properties of the Cocoyol endocarp to unveil structure-property relationships that improve our understanding of the hard fruit shell.

## Results

### Microstructure

**Figure 1:**
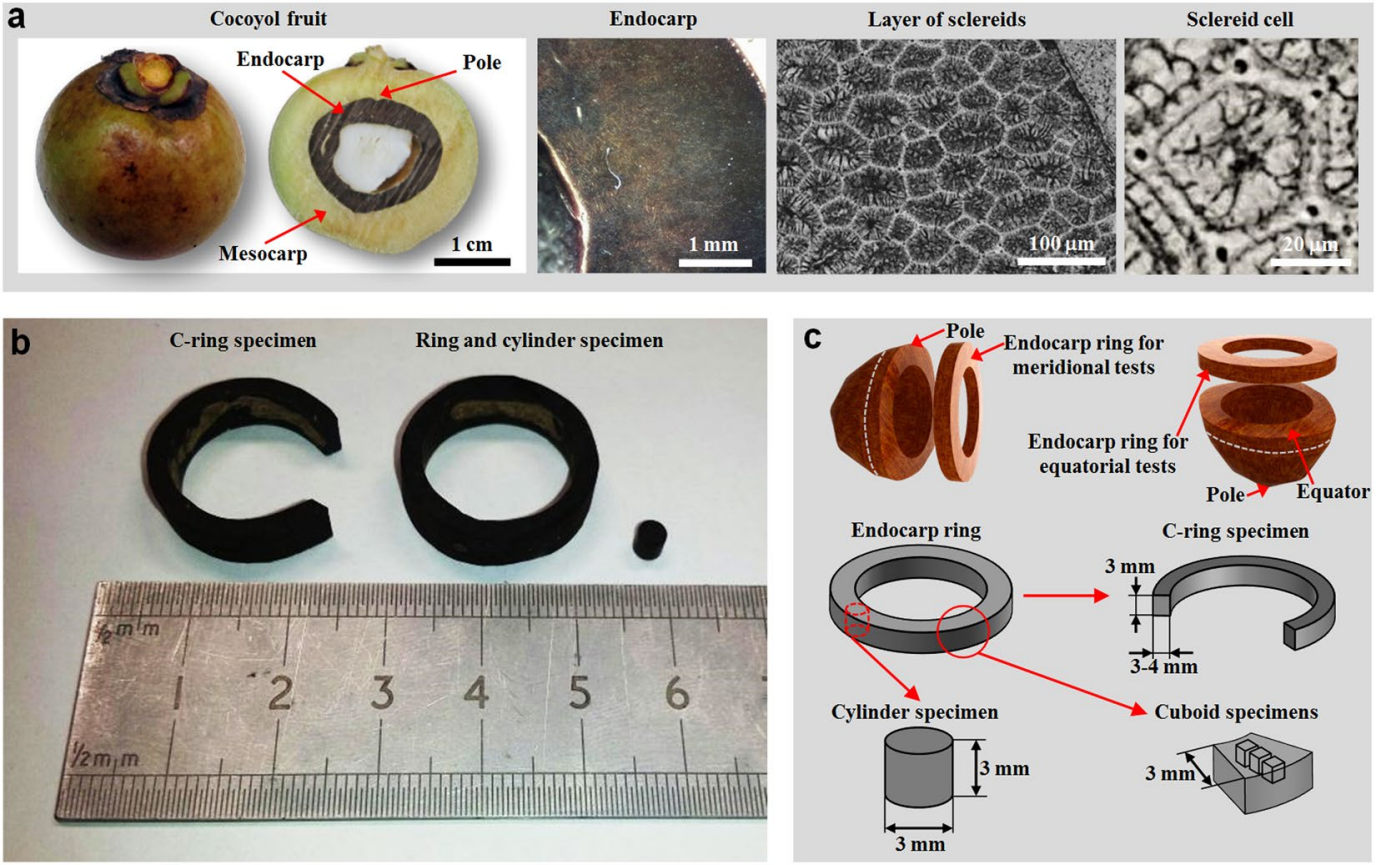
Cocoyol fruit and endocarp specimens. (**a**) Hierarchical structure of Cocoyol endocarp. (**b**) Specimens for microscopy analysis and mechanical testing. (**c**) Geometrical details of specimens.

The microstructure of the Cocoyol endocarp was investigated by optical and SEM images and X-ray micro-CT scan. We start by analysing the microstructure in the radial direction using optical microscopy. Then we utilize micro-CT and SEM images to analyse the 3D shape and orientation of cells. Figure 1a shows the hierarchical structure of the Cocoyol fruit at different length scales. The cross section of the fruit shows its constituent parts. The cross section of the endocarp shows that its microstructure consists of sclerenchyma cells. Specimens used for microscopy studies and mechanical characterizations obtained from the endocarp are shown in Fig. 1b,c and detailed in the Methods section, where the number of specimens used for each testing condition is also specified. The average density of cylindrical samples is 1.255 ± 0.028 g/cm^3^ as detailed in the Methods section.

**Figure 2:**
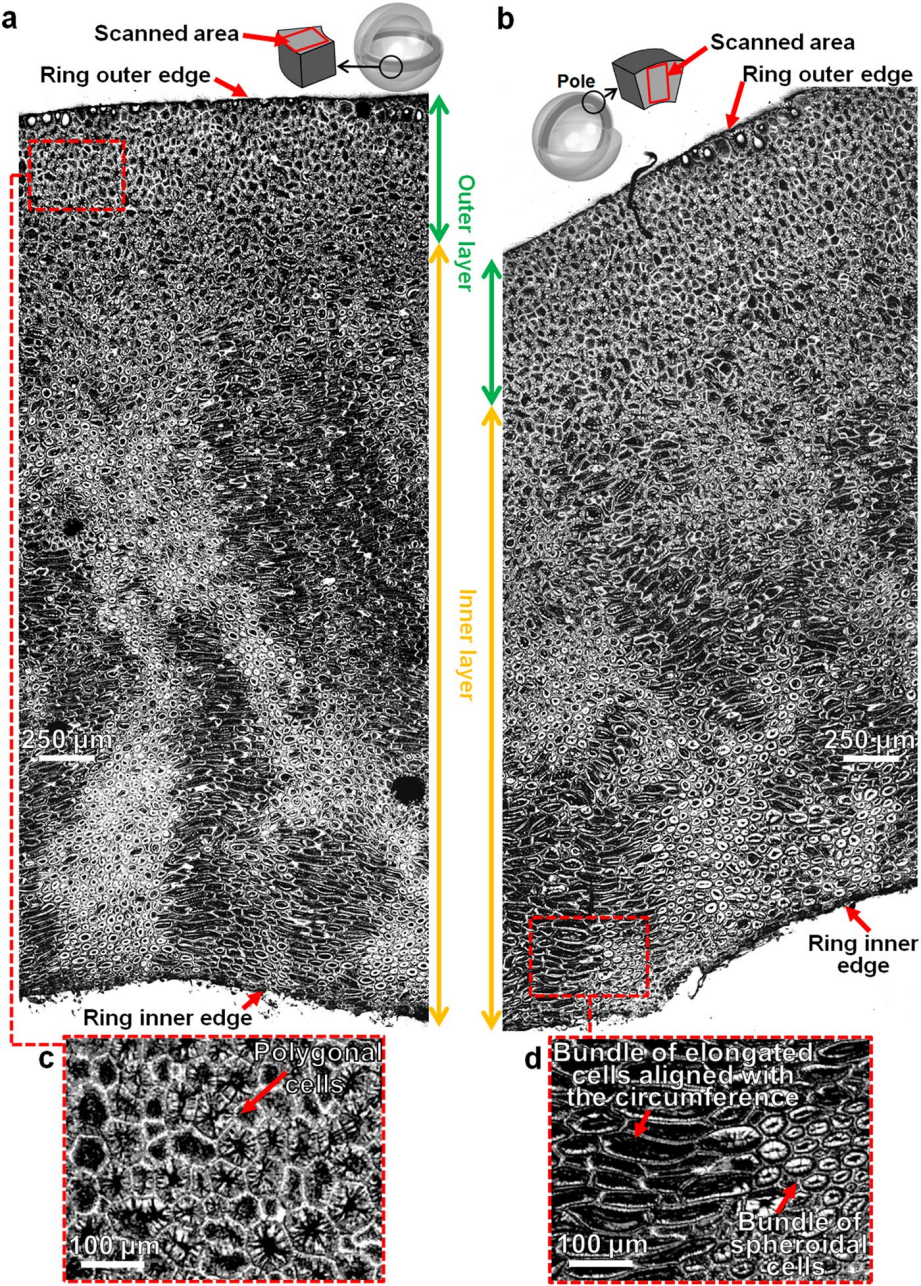
Optical micrographs of Cocoyol fruit endocarp. (**a**) Cross section of fruit endocarp in equatorial plane. (**b**) Cross section of fruit endocarp in meridional plane. (**c-d**) Close-up views.

**Figure 3:**
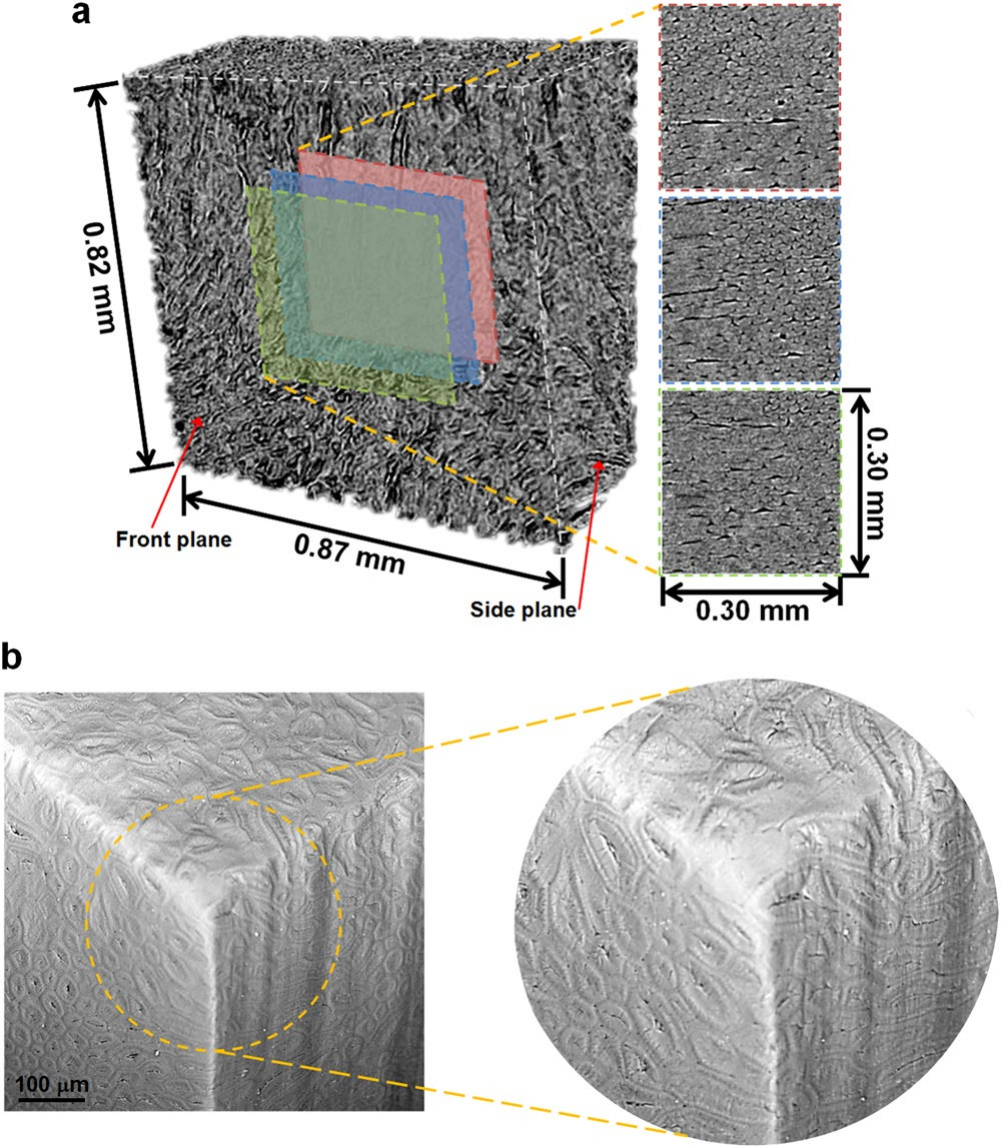
Representative 3D micro-CT and SEM images of Cocoyol endocarp. (**a**) 3D micro-CT scan; slices were obtained at various depths, from a central sub-volume of the scanned sample. The brighter contrast indicates distribution of cells and darker regions indicate cell lumina and space between cells. (**b**) SEM images of the edges of a cuboid specimen showing the 3D shape of elongated sclereids.

Cross sections of the fruit endocarp were polished and then investigated using optical microscope at two different magnifications, as detailed in the Methods section. Typical optical images of cross sections in both equatorial plane (Fig. 2a) and meridional plane (Fig. 2b), show that there is little variation of cells distribution among these two directions. The microstructure consisting of sclerenchyma cells does not vary systematically with the radial position; however, two main layers of cells (outer layer and inner layer in Fig. 2a,b) without clear boundary between them can be identified across the thickness of the ring in the radial direction. The transition between these two layers is gradual; however, some distinctive features can be identified for each layer. In the outer layer, which is near the external edge, the cells have polygonal shape (Fig. 2c) and seem to be densely packed and compactly arranged with no obvious space between them; this layer is around 20% of the total thickness of the ring specimen along the radial direction. Below the outer layer, at the top part of the inner layer, there is an area which is around 20% of the ring thickness, where cells do not have a main single shape, i.e., some cells have polygonal shape, some cells are circular in shape, and some others have a shape of an irregular polygon with curved sides. In the bottom part of the inner layer which is around 60% of the ring thickness, cells are found to be mainly sclereids, with thick strongly lignified secondary wall^51^ and small hollow space, which are either circular in shape (spheroids in Fig. 2d) or elongated cells parallel to the ring circumference (elongated cells in Fig. 2d). Some of the spheroid cells in the inner layer are elongated cells perpendicular to the scanned area, as shown by SEM and micro-CT images in Fig. 3. Both spheroids and elongated cells appear to be grouped in bundles, which is more obvious in the area closer to the inner edge, where it seems that the bundles of elongated cells are better aligned with the ring inner edge and the length of these cells is larger than those cells in the top part of the inner layer.

From the 2D micrographs, the measured diameter of the cells is between 20-60 microns and they have lengths of 1-8 cell diameters. It can be seen in Fig. 2a that the elongated sclereids are not perfectly straight but slightly curved. Also, groups of spheroid cells are observed, which may be elongated cells perpendicular to the cross section (Fig. 2d). A similar microstructure with a similar size range has been reported for Macadamia shell^34–36^; however, that type of shell contains more hollow space, i.e., cell cavities and intercellular space.

Cuboids specimens with a volume of ~1 mm^3^ were used for micro-CT scanning and calculation of density at three different locations across the thickness of the ring in the radial direction (Fig. 1c); volume and weight of cuboids were evaluated using micro-CT and an analytical balance, respectively. One cuboid per location obtained from a single nut was used. The distance between cuboid centres was ~1 mm with a separation between cuboids of ~0.1 mm (Fig. 1c). The representative density of the selected sample near the inner edge (location 1), in the middle (location 2), and near the outer edge (location 3) was 1.122 ± 0.135 g/cm^3^, 1.225 ± 0.214 g/cm^3^, and 1.27 ± 0.19 g/cm^3^, respectively. Here, we report one measured density per location. The three cuboids were obtained from the same slice of a single ring specimen, as shown in Fig. 1c. This apparent gradual increase of density from the inside to the outside surface of the endocarp, with a gradient of density of 0.103 g/cm^3^/mm from location 1 to location 2, and 0.045 g/cm^3^/mm from location 2 to location 3, suggests that the material structure results in a functionally graded material. It is noted that the measured difference of density between cuboids is within the range of experimental error due to the resolutions of the analytical balance and the corresponding volume measurement, and further studies are needed to correlate the density gradient to the mechanical property gradient; however, this density gradient trend is consistent with the stiffness gradient and cell packing discussed later.

As mentioned before, it seems that several cells are isodiametric or spheroids (Fig. 2d); however, a typical 3D micro-CT scan (Fig. 3a) reveals elongated cells with various orientations. It is noted that although micro-CT resolution is insufficient to quantitate certain cell features such as primary and secondary wall thicknesses, it is sufficient to qualitatively distinguish the 3D cell shape and orientation. If we focus on the corners of the scanned volume, it is revealed that the cellular structure is indeed a complex entanglement of bundles of elongated cells (Fig. 3a). For instance, a cell in the front plane looks like a polyhedral cell while the same cell in the side plane looks like an elongated cell. This 3D distribution is confirmed by SEM images, in which the corners of a cuboid specimen (Fig. 3b) show the 3D shape of elongated cells in two perpendicular adjacent planes.

### Mechanical properties by compression and C-ring tests

**Figure 4:**
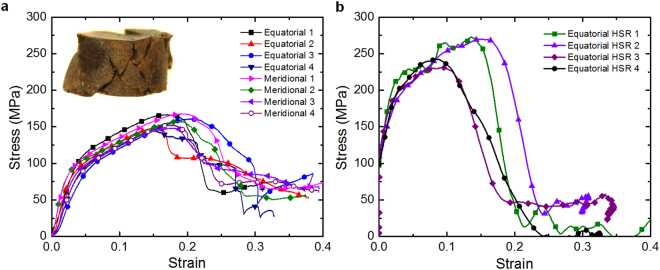
Typical uniaxial compression true stress-true strain curves. (**a**) Quasi-static compression tests. (**b**) High strain-rate (HSR) compression tests (at ∼10^3^ s^−1^).

Figure 4a shows true stress-true strain curves for cylinder specimens under quasi-static compression. Specimens have a height to diameter ratio of 1:1 (height 3.082 ± 0.069 mm and diameter 3.095 ± 0.042 mm). It can be seen that the maximum stress levels are in the range of 140–170 MPa (with an average of 156 MPa) prior to specimen failure at around 0.18 strain. Two distinctive regimes are observed before the peak stress, which are an elastic regime up to a strain of ∼0.03 with a yield stress of around 80 MPa, and a plastic regime with strain hardening up to failure strain.

**Figure 5:**
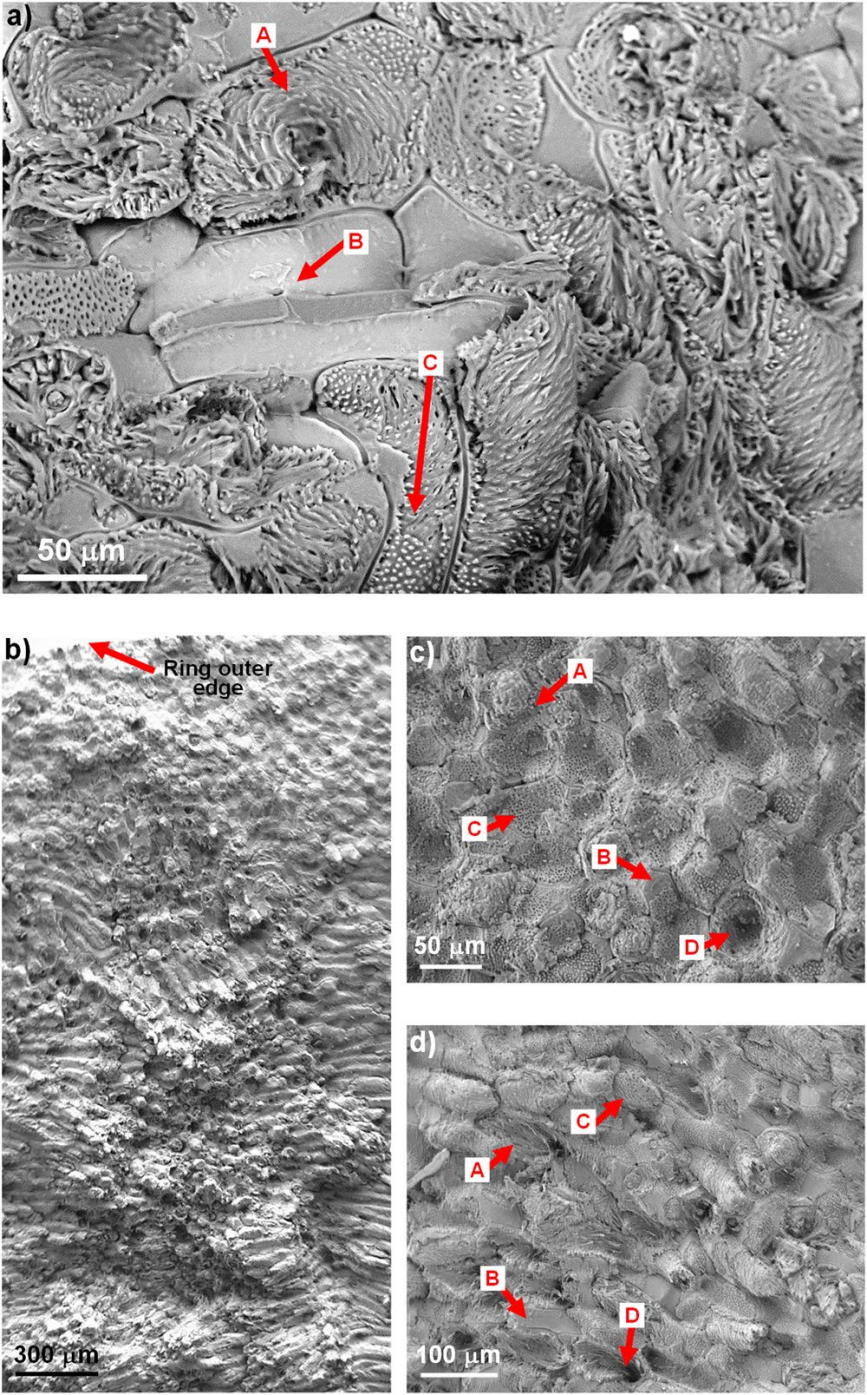
SEM images of crack surfaces. Identified failure mechanisms include A. Cell tearing; B. Middle lamella breakage; C. Primary cell wall breakage; D. Pull-out of elongated cells. (**a**) Crack surface after quasi-static compression test. (**b**) Crack surface after C-ring test. (**c**) Close-up image of surface near the outer edge after C-ring test. (**d**) Close-up image of surface near the inner edge after C-ring test.

Figure 5a shows an SEM image of the crack surface of a sample under quasi-static compression. Different energy-absorbing mechanisms can be identified from this image: A. Cell tearing which occurs when the crack runs through a cell or a cell bundle; B. Middle lamella breakage which occurs when the primary cell wall remains fairly intact. This resembles somehow delamination between cells; C. Primary cell wall breakage, which occurs when primary wall is severely damaged. This failure mechanism (C) is interesting because it seems that the middle lamella remains fairly intact, indicating that the intercellular bonding is very strong. It can also be observed in Fig. 5a that failure mechanism (A) includes the breakage of the multilayered secondary wall, which comprises the breakage of individual layers; some delamination between individual layers may also be included in this failure mechanism. It is also noted that because of the difficulty to differentiate between primary wall and the outer layers of the secondary wall, failure mechanism (C) may include in some cases the breakage of some external layers of the secondary wall. From Fig. 5a, it seems the different energy-absorbing mechanisms are determined by the cell orientation with respect to the front of a propagating crack.

True stress-true strain curves from high strain-rate compression tests (strain rate ∼10^3^ s^−1^) are shown in Fig. 4b. It can be seen that yield strength is reached at about 160 MPa and peak stress levels are in the range of 230–270 MPa (with an average of 253 MPa), which are ∼90% and ∼60% higher than the observed yield stress and peak stress in quasi static test, respectively; however, the endocarp becomes less ductile and failure strain occurs at approximately 0.12 strain, which is about 33% lower than that observed in quasi-static tests. The mechanical properties obtained from compression tests are shown in Table 1. Toughness (work of fracture) is defined as the area under the stress-strain curve up to failure.

**Table 1:** Mechanical properties of Cocoyol endocarp.

Test (direction)	Young’s modulus *E* (GPa)	Yield strength ${{\boldsymbol{\sigma }}}_{{\bf{y}}}$ (MPa)	Maximum stress ${{\boldsymbol{\sigma }}}_{{\boldsymbol{\max }}}$ (MPa)	Maximum strain ${{\boldsymbol{\varepsilon }}}_{{\boldsymbol{\max }}}$	Toughness (kJ/m^2^)
Compression (Equatorial)	3.02 ± 0.57	87.3 ± 12.2	156 ± 10.4	0.17 ± 0.03	20.1 ± 4.8
Compression (Meridional)	3.94 ± 0.81	80.2 ± 2.02	157 ± 9.18	0.19 ± 0.01	20.9 ± 1.5
SHPB (Equatorial)	—	161 ± 14.4	253 ± 20.4	0.12 ± 0.04	24.8 ± 8.4
C-ring (Meridional)	6.08 ± 1.23	—	79.1 ± 14.3	—	—
C-ring (Equatorial)	5.26 ± 1.29	—	107 ± 18.6	—	—

The values of Young’s modulus and maximum tensile stress obtained from C-ring tests are depicted in Table 1. Figure 5b–d shows SEM images of fracture surfaces of the C-ring specimen in the equatorial direction. The fracture surface is coplanar but rough. Some of the failure mechanisms identified are similar to those observed in compression, i.e., cell tearing (A), middle lamella breakage (B) and primary cell wall breakage (C). Pull-out (D) of elongated cells is also observed. In areas where cells are mainly polyhedral near the outer edge (Fig. 5c), mechanisms B and C are dominant; the crack runs around the cell following an intercellular path, i.e. the cells are not severely damaged, and the main damage is observed at the primary cell wall or in the middle lamella. In areas where elongated cells are grouped into bundles (near the inner edge and in the middle section, Fig. 5d), mechanisms A and D are dominant. It can be seen that the pull-out (D) of elongated cells (Fig. 5d) also includes tearing of primary wall and the crack runs through the cells following a transcellular path. It seems that the damage mechanism is closely related to the cells orientation. The combinations of these mechanisms lead to extended crack paths and thus an increase in toughness is developed.

### Mechanical properties by nanoindentation and Vickers hardness

**Figure 6:**
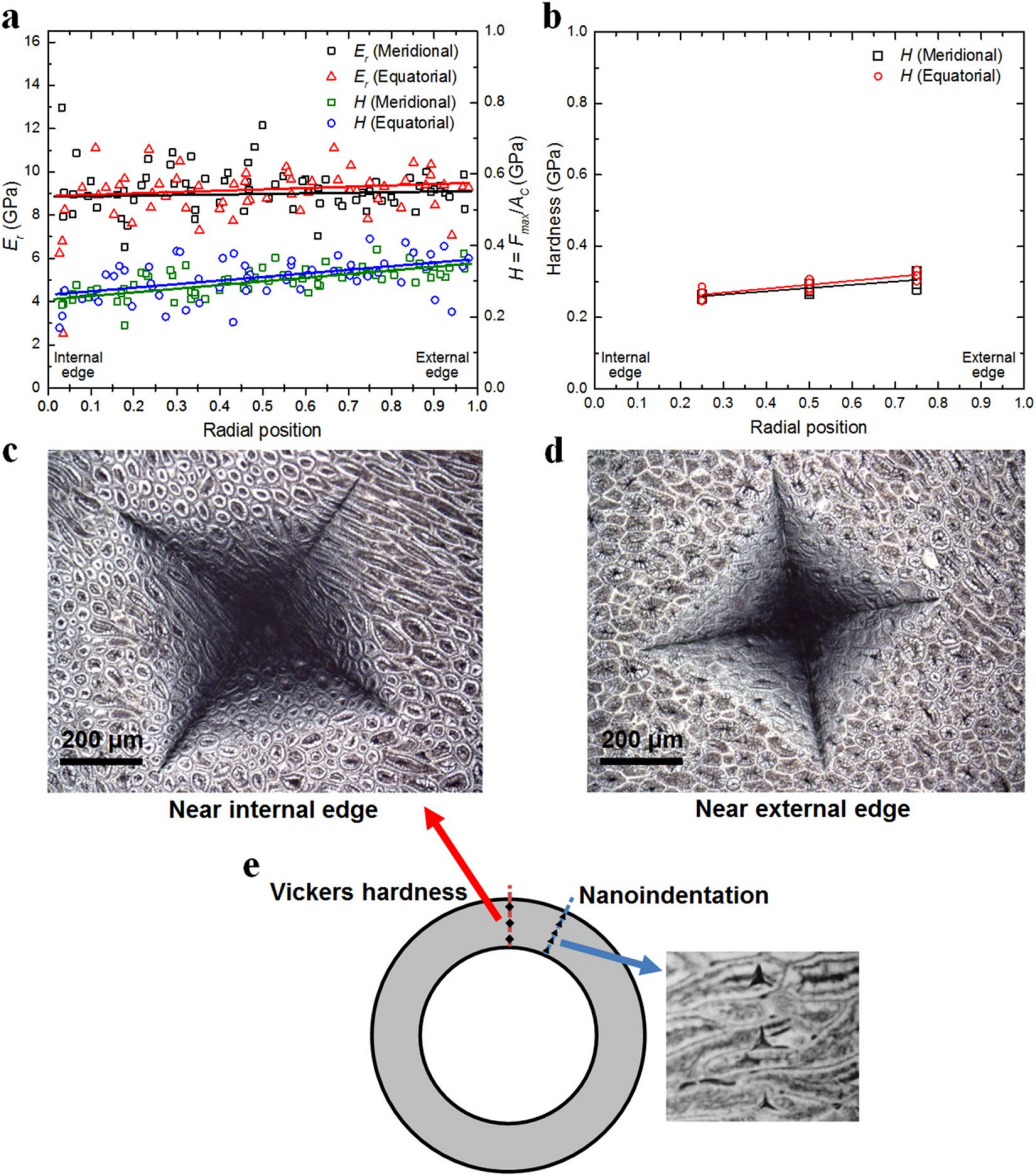
Nanoindentation and Vickers hardness results. (**a**) Reduced elastic modulus *E*_*r*_ and hardness *H* of the endocarp measured from the internal edge (IE) to external edge (EE) for meridional and equatorial specimens at a maximum load of 100 mN (nanoindentation). (**b**) Vickers hardness results. (**c-d**) Typical Vickers indentation marks. (**e**) Zones in the ring specimen for nanoindentation and Vickers hardness tests.

Figure 6a shows the results from nanoindentation with the maximum applied normal load being 100 mN. Negligible influence was observed on the measured mechanical properties for different maximum load levels (ranging from 10 mN up to 500 mN). The measured reduced elastic modulus *E*_*r*_ and hardness *H* at different positions are shown for both meridional and equatorial orientations in Fig. 6a. It is clear from Fig. 6a that there is little variation of the properties among these two directions, which is consistent with the obtained similar mechanical properties in the compressive tests in both directions. This indicates that the structure is fairly homogenous along the circumference of the ring samples. It can also be seen that both the reduced elastic modulus and the hardness increase slightly towards the outer edge of the Cocoyol ring specimen. This increase is more obvious for the hardness value. The gradients of *E*_*r*_ and *H* were determined from the slope of the linear fits to the data. The gradients of the *E*_*r*_ were 89.3 MPa/mm and 204.7 MPa/mm for the meridional and equatorial directions, respectively. The gradients of the *H* were 34.2 MPa/mm and 35.8 MPa/mm for the meridional and equatorial directions, respectively.

Figure 6b shows Vickers hardness test results. For comparison with nanoindentation results, Vickers hardness HV was converted from the conventional units (kgf/mm^2^) to MPa through the conversion factor of 9.806 MPa mm^2^ kgf^−1 ^^52^. It can be seen that hardness values are in the range of 0.25–0.31 GPa, and are consistent with the results obtained from nanoindentation. Moreover, a slight increase of hardness from inner edge to outer edge can be observed for the Vickers hardness as well for both meridional and equatorial directions. Typical Vickers indentation marks are shown in Fig. 6c-d. The values of the gradients of hardness were determined from the slope of the linear fits to the data, as 30.5 MPa/mm and 37.2 MPa/mm for the meridional and equatorial directions, respectively.

## Discussion

The mechanical results show that Cocoyol endocarp is indeed a very hard and tough material. Stress-strain curves showed two distinctive regimes, which are an elastic regime, and a plastic regime with strain hardening up to failure strain. The elastic behaviour can be understood as stretching and bending of cell walls since the fruit endocarp can also be seen as a pseudo closed-cell rigid foam with thick walls. The plastic behaviour can be understood as a combination of different energy absorbing mechanisms including cell crushing, middle lamella breakage, primary wall breakage and cell tearing (secondary wall breakage), as shown in Fig. 5a. As the specimen densifies with compressive strain greater frictional energy dissipation is present. It is noted that after the failure strain, a non-catastrophic drop in the stress (softening) is observed, i.e. the specimen is capable to carry load of around 70 MPa up to 0.4 strain. This can be explained by the fact that several shear-type cracks intersecting each other were developed during the loading process in the yielded specimen indicated by inclined fracture lines at several locations, shown in the inset of Fig. 4a. These results indicate that the entanglement of elongated cells prevents crack localization and promotes more globalized plastic deformation. This in turn generates a slowdown of crack propagation by redirecting crack propagation path or arresting the crack propagation at some locations, which results in a large amount of energy being dissipated in the development of cracks. True stress-true strain curves from high strain-rate compression tests showed that both yield strength and peak stress levels are higher than those observed in the quasi static test; however, the endocarp becomes less ductile. This is the typical strength-ductility trade-off mechanism observed in other materials deformed at high strain rates^53^. These results clearly show a strain-rate dependence of the fruit shell, which may be explained by the strain rate sensitivity of cell material due to viscosity and by localized deformation resulting in subsequent hardening and less ductility.

The complex microstructure of this fruit shell consists of two main layers, i.e., one thin and harder outer layer near the external edge with cells having polyhedral shape, and a thick inner layer of entangled bundles of elongated sclereid cells with longer cells near the inner edge. This multi-layer arrangement with hardness decreasing from the outer layer to the inner layer may work as a protection against predators, such insects trying to penetrate the endocarp for oviposition^54^, small mammal herbivores and birds^55^. *Acrocomia aculeata* fruits may also be subjected to impact forces when they are transported by birds and some of them fall to the ground^55,56^. In this case, the whole fruit would be subjected to uniaxial compression, in which, geometrical factors, such as the variation of endocarp thickness may influence the toughness of the whole shell by stress concentrations in the areas with reduced thickness; however, we found that Cocoyol endocarp thickness is fairly homogenous (3–4 mm). The multi-layer arrangement also works as functionally graded material, where the hardness and elastic modulus gradients promote load transfer and a gradual stress redistribution, which in turn produces a more uniform internal stress distribution and greater area of plastic straining in the radial direction^57^; this suggests that the gradients of *E* and *H* values are optimized towards preventing crushing. Spatial gradients of mechanical properties in plants are an evolutionary adaptation strategy for resisting severe mechanical forces by accommodating larger deformations to lower stress concentrations^58^. For example, the culms of the giant reed (*Arundo donax*) exhibit gradual changes in stiffness within parenchymatous and sclerenchymatous tissues; however, the cellulose microfibril orientation does not show a gradual transition, which may be explained as an evolutionary strategy to compromise between avoiding stress discontinuities and optimizing bending stiffness of the entire culm^58,59^. Moso bamboo (*Phyllostachys pubescens*) culm exhibits a radial gradient in tissue density and vascular bundle volume with denser tissue towards the outer part of the culm, which results in an increase of axial stiffness from inside towards the external surface^2,58^. For the Cocoyol endocarp, the elastic modulus and the hardness increase towards the outer edge of the ring specimen. These gradients may be explained by the different cell shapes and distributions observed in the two main layers identified in Fig. 2a,b and the apparent gradual increase of density from the inside to the outside surface of the fruit shell; the hardness will be higher in the outer layer because there is a denser packing of cells due to their polyhedral shape (Fig. 2c). The same trend is observed for both directions. It is noted that Fig. 2a,b suggest that there is less intercellular substance in the outer layer (middle lamella or compound middle lamella), which may also explain the increase of hardness in the vicinity of the outer edge; however, the thickness of intercellular material was not measured in the current study.

In the inner layer, the cell bundles seem to be randomly oriented; however, closer observation revealed that some patterns in the form of pillars of elongated cells aligned with the circumference are present in some areas near the inner edge (see bottom of inner layer in Fig. 2a). This results in bundles of different sizes and alternate orientation like an entanglement of bundles. This type of arrangement provides resistance against hoop stresses at the internal edge located beneath a loading point, where cracks tend to appear when the whole fruit shell is subjected to uniaxial compression^60^. The way that the pillars are arranged shows that a crack must follow a tortuous path in order to propagate and this will require more energy for the crack to develop.

**Figure 7:**
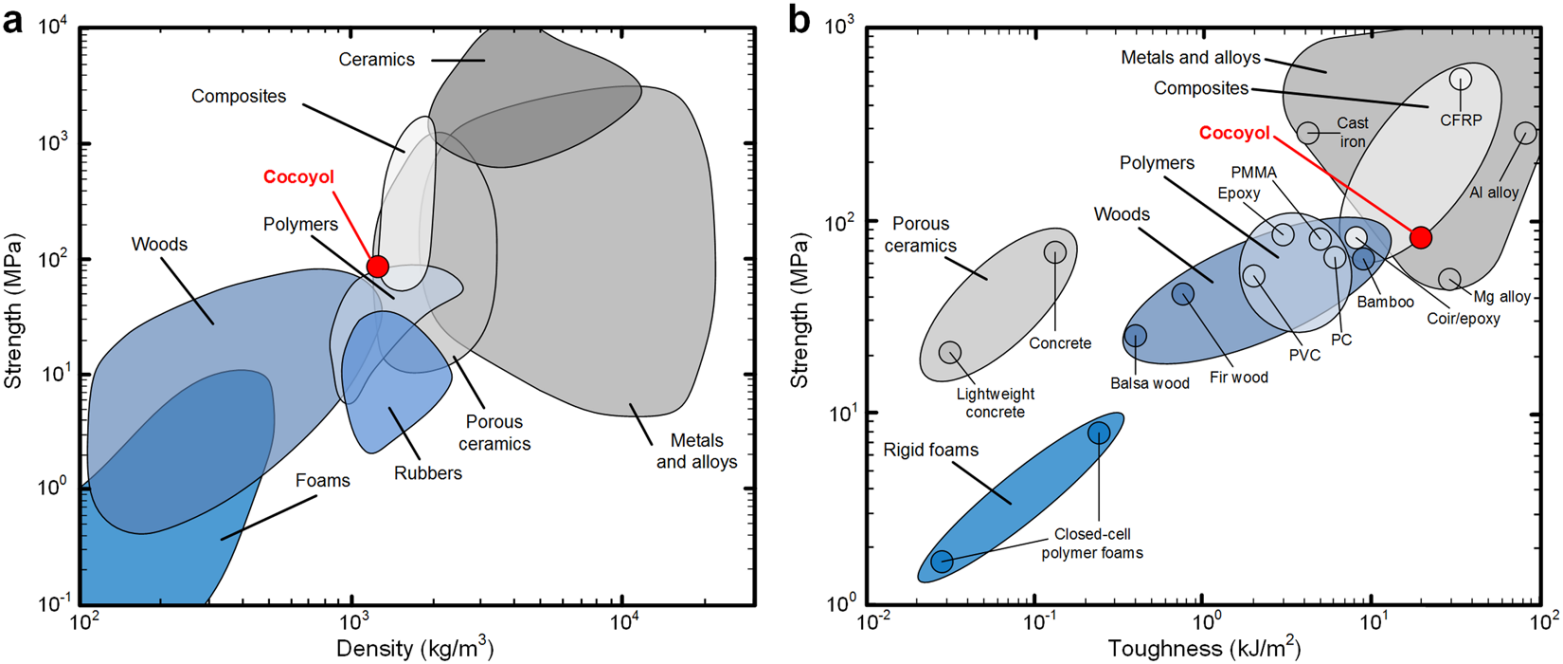
Ashby charts for different types of materials including the Cocoyol endocarp. (**a**) Strength versus density (Adapted from: http://www-materials.eng.cam.ac.uk/mpsite/interactive_charts/). (**b**) Strength versus toughness (From data available in the literature: rigid foams^61^, concrete^62^, woods^2,63^,^64^, polymers^65^, metals and alloys^66–68^, composites^69^,^70^).

These findings demonstrate that structure-property relationships make this material hard and tough. Figure 7 shows Ashby charts for compressive strength versus density and compressive strength versus toughness for different materials. It is noted that the toughness (work of fracture) presented in Figure 7b corresponds to the area under the stress-strain curve up to failure in quasi-static uniaxial compressive test of material data available in the literature. The Cocoyol endocarp shows a superior performance when compared with other natural wood materials. Through evolutionary development, the properties of Cocoyol endocarp are even comparable with those of synthetic materials designed for structural purposes such as composites (Fig. 7).

Table 2 shows a comparison of the mechanical properties of various fruit and nut shells. Since most of the available data for these types of shells is from indentation tests, only values of *E*_*r*_ and *H* are presented for all the shells. It can be seen that the Cocoyol shell exhibits excellent mechanical properties overall when compared to the other shells. Based on the values of hardness, toughness (work of fracture) and failure strain exhibited by the Cocoyol shell, one can hypothesize that the mechanical properties of the Cocoyol endocarp may be an evolutionary strategy to compromise between having higher hardness and optimizing toughness of the whole shell.

**Table 2:** Mechanical properties of various fruit and nut shells.

Fruit shell	Reduced modulus *E*_*r*_ (GPa)	Hardness *H* (MPa)	Yield strength ${\sigma }_{y}$ (MPa)	Density (g/cm^3^)
Cocoyol (*Acrocomia mexicana*)	9.1 ± 1.28^a^	310 ± 59^a^ 290 ± 25^b^	87.3 ± 12.2	1.25 ± 0.03
Macadamia (*Macadamia ternifolia*)	8.5 ± 0.08^a^^26^	540 ± 40^a^^26^ 180 ± 30^b^^34^	84 ± 9^35^	1.27^34^
*Scheelea sp*.	3.80 ± 0.99^b^^71^	271.5 ± 74.7^b^^71^	—	—
Oil palm tree (*Elaeis guineensis*)	2.46 ± 1.04^b^^71^	126.3 ± 20.6^b^^71^	12 ± 1.98^72^	1.14^72^
Coconut palm tree (*Cocos nucifera*)	3.52 ± 0.56^c^^73^	116 ± 10^c^^73^	—	1.05–1.20^74^

^a^Nanoindentation hardness. ^b^Vickers hardness. ^c^Spherical indenter.

In Conclusion, the study of the microstructure and mechanical properties of Cocoyol palm fruit (*Acrocomia mexicana*) has provided new insights into structure-property relationships of natural fruit shell materials that produce outstanding hardness and toughness, which inspires the microstructural design of man-made materials. These findings show that the Cocoyol endocarp is a functionally graded material with two distinctive layers along the radial directions. The outer layer is formed by polygonal densely-packed cells while the inner layer is formed by bundles of elongated cells. The apparent gradual increase of density from the inside to the outside surface agrees with the gradients of elastic modulus and hardness, which increase linearly from the inner edge to outer edge. Different failure mechanisms including cell tearing (secondary cell wall breakage), middle lamella breakage, primary cell wall breakage and cell pull-out were identified in the crack surface images. These mechanisms seem to be cell-orientation dependent and they promote more globalized plastic deformation, which in turn generates a slowdown of crack propagation and results in more energy dissipation. The endocarp also is a strain-rate sensitive material that can absorb more energy at higher rates.

Our results warrant further research of the Cocoyol shell from a multidisciplinary approach at lower length scales to reveal more underlying toughening mechanisms of the hierarchical structure. These studies should include measurement of mechanical properties of individual structural constituents of cell and multi-scale numerical modelling to fully understand the toughening mechanisms governing the macroscopic mechanical properties of this hard and tough shell. For now, in light of these findings, it is reasonable to confirm that the Maya legend was true in the sense that the Cocoyol fruit is indeed hard and tough.

## Methods

### Material

We harvested Cocoyol palm fruit racemes (*Acrocomia mexicana* Karw. ex Mart.^38^) from trees located approximately 50-km east from Merida, Yucatan, Mexico. Fruits of similar size were selected (Fig. 1a) and dried at 80 °C in an oven for 48 h. Previous work on compression of whole Cocoyol fruit showed that although a slightly better performance is observed for the non-dried fruits, drying condition does not affect substantially the mechanical performance of the fruit^50^. After drying, exocarp and mesocarp were removed. The endocarp was used to obtain specimens for mechanical testing and microscopy analysis (Fig. 1b) with dimensions shown in Fig. 1c. From a fruit, a single endocarp ring was obtained for either equatorial direction tests or meridional (polar-polar) direction tests (Fig. 1c). From an endocarp ring, either cylinder specimens or C-ring specimens were obtained. Specimens were examined to ensure that no existing visible cracks or cracks formed during drying were present prior to testing.

### Compression testing

Compression tests were performed on cylinder specimens (Fig. 1c) with a loading frame (Criterion 43, MTS, USA) at a crosshead speed of 0.1 mm/min. Samples were obtained from endocarp rings cut at different orientations (meridional or equatorial, Fig. 1c), exhibiting a height to diameter ratio of 1:1, with average height of 3.082 ± 0.069 mm and diameter 3.095 ± 0.042 mm over 12 samples. The average density of this material (1.255 ± 0.028 g/cm^3^) was obtained by measuring the weight using an analytical balance and the corresponding volume based on the amount of displaced water. The load was measured with a 50-kN load cell attached to the loading frame. The displacement was acquired using a linear variable differential transducer (LVDT). Considering the low expansion of the compressed specimen due to its cellular structure, we recorded the deformation during the loading process through taking a set of photos with respect to displacement of the crosshead, at intervals of 2.5% strain and synchronized the specimen expansion profile with the recorded compressive load. This enabled the calculation of true stress and true strain. High strain-rate compression tests were performed on cylinder samples (Fig. 1c) using a 15 mm diameter split Hopkinson pressure bar (SHPB). The device bars were made of aluminium alloy 7075 both 1500 mm in length.

### Nanoindentation and Vickers hardness tests

Nanoindentation tests were performed to characterize the mechanical behaviour of the endocarp of Cocoyol. Ring samples were cut from a complete fruit either along meridional or equatorial direction, yielding a circular ring with thickness between 3–4 mm (Fig. 1b). The inner and outer radii were about 7 mm and 10 mm, respectively. The samples were then prepared using sequential grinding and polishing steps with final polishing using 1 μm diamond suspension. A Nanoindenter (G200, Agilent, USA) with a Berkovich tip (TB22122, MICRO STAR, USA), which has a three-side pyramid geometry with a tip radius of about 20 nm, was employed. Prior to the nanoindentation test, the system was calibrated using a fused silica standard. The load and depth of penetration were recorded under the load-control mode. Load-indentation depth curves were used to extract mechanical properties, including reduced elastic modulus and hardness, according to the approach developed by Oliver and Pharr^75^.

The nanoindentation tests were conducted on two polished ring samples of different cutting orientation, meridional and equatorial. As shown in Fig. 6e (top view of the ring sample), eight zones were chosen for indentation tests on one sample. At each individual zone, the first indentation position is about 50 μm from the inner edge (where radial position *d*/*D* = 0), with the subsequent indentation positions moving radially a distance *d* towards the outer edge (where *d*/*D* = 1). *D* is the width of the sample ring. The distance between two adjacent indentation positions is about 60 μm, thus 50 to 70 indentations were achieved in each zone.

The hardness is tested with a Vickers hardness tester (LV700AT, LECO, USA). The Vickers tip of the indenter was loaded to a peak force of 10 kgf, then hold at constant load for 15 s. For each sample, the tests were also carried out at eight zones (Fig. 6e); in each of them three indents were made on different points: inner, middle and outer (approximately 0.25*D*, 0.5*D* and 0.75*D* to the inner edge).

### C-ring

To further quantify the mechanical properties, 12 C-ring specimens were prepared for compression tests. All experiments were carried out with the loading frame (Criterion 43, MTS, USA) at a loading speed of 0.5 mm/min. Young’s Modulus and the ultimate tensile strength were obtained from the measured load-deflection curves until the fracture occurs. The relationship between the applied load $P$ and the deflection $\delta$ is given by

1 $$P=\frac{4b}{3{\rm{\pi }}}\frac{E{t}^{3}}{{(2{r}_{0}-t)}^{3}}\delta $$

where $b$ is the thickness of the specimen, $t$ is the ring width at the centre, ${r}_{0}$ is the outer radius and $E$ is the Young’s modulus. Based on beam curved theories, the ultimate tensile stress on the outer surface where the fracture initiates is expressed as

2 $${\sigma }_{max}=\frac{2P(3{r}_{0}-2t)}{b{t}^{2}}$$

From the above equations, the Young’s modulus and the maximum tensile stress were calculated from the experimental data^35^.

### Microscopy

Surface examination of the samples was performed using an optical microscope at two different magnifications. Scanning electron microscopy (SEM) was also performed (Carl Zeiss AG EVO 50; JEOL Neoscope JCM 6000) on polished specimens and fracture surfaces. Polished specimens were prepared using sequential grinding and polishing steps with final polishing using 1 μm diamond suspension.

### CT-Scan

Cuboid specimens with volume of ~1 mm^3^ were cut from one Cocoyol endocarp wall and properly polished before the scanning using micro-CT (Xradia MicroXCT-400 Micro-Computed Tomography, Carl Zeiss, Germany) with an X-ray energy of 60 kV and submicron resolution (<1 μm). To calculate the density of the cuboids, their apparent volume was measured from CT scans and weight was measured using an analytical balance NUWEIGH XA82/220/X with a readability for 0.01 mg.
